# Supplementary material for: The Impact of Mode of Birth, and Episiotomy, on Postpartum Sexual Function in the Medium- and Longer-Term: An Integrative Systematic Review
Source: Int J Environ Res Public Health. 2023 Mar 24;20(7):5252. doi: 10.3390/ijerph20075252 (PMC10094321; doi:10.3390/ijerph20075252)
Supplement: Supplementary file 1 [file ijerph-20-05252-s001.zip › ijerph-2225179-supplementary.pdf]

Supplementary Materials

Table S1. Domains and options for quality assessment using NOS

Domains	
**Selection**	Options
Representation of exposed cohort	a) truly representative of the average pregnant woman in the community ★<br>b) somewhat representative of the average pregnant women in the community ★<br>c) selected group of users<br>d) no description of the derivation of the cohort
Selection of the non-exposed cohort from same source as exposed cohort	a) drawn from the same community as the exposed cohort ★<br>b) drawn from a different source<br>c) no description of the derivation of the non exposed cohort
Ascertainment of exposure	a) secure record (eg surgical records) ★<br>b) structured interview ★<br>c) written self report<br>d) no description
Outcome of interest was not present at start of study	a) yes ★<br>b) no
**Comparability**	
Adjustment for most important confounder	Study controls for specific mode of birth ★
Adjustment for any other confounder	study controls for any additional factor ★
**Exposure**	
Assessment of outcome	a) independent blind assessment ★<br>b) record linkage ★<br>c) self report<br>d) no description
Follow up long enough for outcome to occur	a) yes (≥ 6 months postpartum) ★<br>b) no
Adequacy of follow up (follow-up rate)	a) complete follow up - all subjects accounted for ★<br>b) subjects lost to follow up unlikely to introduce bias - small number lost - ≥ 80% follow up, or description provided of those lost ★<br>c) follow up rate < 80% and no description of those lost<br>d) no statement

Table S2. scoring system for quality assessment using NOS

Domains	Number of stars for quality rating		
	Poor	Fair	Good
Selection	0 / ★	★★	★★★ / ★★★★
Comparability	0	★ / ★★	★ / ★★
Outcomes	0 / ★	★★ / ★★★	★★ / ★★★

Table S3. studies that were excluded after reading full text and reasons for exclusion

**Paper**	**Reason for exclusion**
Abbaspoor, Z., Moghaddam-Banaem, L., Ahmadi, F., & Lili, A. K. (2016). Postnatal Sexual Concerns in Relation to Choice of Delivery Mode among Iranian Women: A Qualitative Content Analysis. Journal of Midwifery & Reproductive Health, 4(2), 613–621.	Modes of birth not specified
Abidi I, Bettaieb H, Frikha M, Halouani S, Mbarki W, Boufarguine R, et al. Sexual dysfunction and satisfaction in Tunisian couples during postpartum: A prospective cohort study. Journal of Sexual Medicine. 2022;19(5):S229.	Modes of birth not specified
Ahmad Shirvani M, Bagheri-Nesami M, Bavand Mazandaran M. Women's sexual dysfunction during the first year after child birth. International Journal of Gynecology and Obstetrics. 2009;107:S557.	Did not compare modes of birth
Alcalay M, Brecher S, Kalter A, Schiff E, Eisenberg V. Different patterns of pelvic floor dysfunction in forceps and vacuum deliveries. International Urogynecology Journal and Pelvic Floor Dysfunction. 2011;22:S69-S70.	Did not use a validated questionnaire
Alp Yilmaz, F., Avci, D., Arzu Aba, Y., Ozdilek, R., & Dutucu, N. (2018). Sexual Dysfunction in Postpartum Turkish Women: It's Relationship with Depression and Some Risk Factors. African journal of reproductive health, 22(4), 54–63. https://doi.org/10.29063/ajrh2018/v22i4.6	Sexual functioning not measured at ≥ 6 months postpartum
Amiri, F. N., Omidvar, S., Bakhtiari, A., & Hajiahmadi, M. (2017). Female sexual outcomes in primiparous women after vaginal delivery and cesarean section. African health sciences, 17(3), 623–631. https://doi.org/10.4314/ahs.v17i3.4	Sexual functioning not measured at ≥ 6 months postpartum
Aribi L, Ben Houidi A, Masmoudi R, Chaabane K, Guermazi M, Amami O. Female sexuality during pregnancy and postpartum: A study of 80 tunisian women. Tunisie Medicale. 2012;90(12):873-7.	Did not compare modes of birth
Arrigo A, Gritti A, Sosso C, Scatigno AL, Dominoni M, Gariboldi F, et al. Effects of instrumental vaginal delivery on sexuality and pelvic floor: Preliminary experience in a cohort of nulliparous women. Neurourology and Urodynamics. 2021;40(SUPPL 3):S105-S6.	Did not use a validated questionnaire
Banaei, M., Moridi, A., & Dashti, S. (2018). Sexual Dysfunction and its Associated Factors After Delivery: Longitudinal Study in Iranian Women. Materia socio-medica, 30(3), 198–203. https://doi.org/10.5455/msm.2018.30.198-203	Sexual functioning not measured at ≥ 6 months postpartum
Barrett, G., Peacock, J., Victor, C.R. and Manyonda, I. (2005), Cesarean Section and Postnatal Sexual Health. Birth, 32: 306-311. https://doi.org/10.1111/j.0730-7659.2005.00388.x	Did not use a validated questionnaire
Barrett, G., Pendry, E., Peacock, J., Victor, C., Thakar, R. and Manyonda, I. (2000), Women's sexual health after childbirth. BJOG: An International Journal of Obstetrics & Gynaecology, 107: 186-195. https://doi.org/10.1111/j.1471-0528.2000.tb11689.x	Did not use a validated questionnaire
Behzadipour S, Daneshpour M, Damreihani N, Aflatooni L. Sexual satisfaction and intimacy during pregnancy and after childbirth. Sexologies. 2021;30(2):e111-e7.	Did not compare modes of birth
Beucher G. Complications maternelles des extractions instrumentales. Journal de Gynécologie Obstétrique et Biologie de la Reproduction. 2008;37(8, Supplement 1):S244-S59.	Not available in English
Bia F. The influence of birth on the female sexual functioning. Sexologies. 2010;19:S81.	Time when sexual functioning was measured was not specified

Boroumandfar, K., Rahmati, M. G., Farajzadegan, Z., & Hoseini, H. (2010). Reviewing sexual function after delivery and its association with some of the reproductive factors. Iranian journal of nursing and midwifery research, 15(4), 220–223.	Sexual functioning not measured at ≥ 6 months postpartum
Brown, S. and Lumley, J. (2000), Physical health problems after childbirth and maternal depression at six to seven months postpartum. BJOG: An International Journal of Obstetrics & Gynaecology, 107: 1194-1201. https://doi.org/10.1111/j.1471-0528.2000.tb11607.x	Did not use a validated questionnaire
Buhling, K. J., Schmidt, S., Robinson, J. N., Klapp, C., Siebert, G., & Dudenhausen, J. W. (2006). Rate of dyspareunia after delivery in primiparae according to mode of delivery. European journal of obstetrics, gynecology, and reproductive biology, 124(1), 42–46. https://doi.org/10.1016/j.ejogrb.2005.04.008	Did not use a validated questionnaire
Cappell, J., Bouchard, K. N., Chamberlain, S. M., Byers-Heinlein, A., Chivers, M. L., & Pukall, C. F. (2020). Is Mode of Delivery Associated With Sexual Response? A Pilot Study of Genital and Subjective Sexual Arousal in Primiparous Women With Vaginal or Cesarean Section Births. The journal of sexual medicine, 17(2), 257–272. https://doi.org/10.1016/j.jsxm.2019.11.264	Sexual functioning not measured at ≥ 6 months postpartum
Chang SR, Chen KH, Lin MI, Lin WA. Sexual dysfunction through 1 year postpartum following vaginal or caesarean delivery. Journal of Sexual Medicine. 2015;12:351-2.	Poster/conference abstract of paper that was already included
Chayachinda, C., Titapant, V., & Ungkanungdecha, A. (2015). Dyspareunia and sexual dysfunction after vaginal delivery in Thai primiparous women with episiotomy. The journal of sexual medicine, 12(5), 1275–1282. https://doi.org/10.1111/jsm.12860	Did not compare modes of birth
de Lima Holanda, J. B., de Sá Vieira Abuchaim, E., Coca, K. P., & de Vilhena Abrão, A. C. F. (2014). Sexual dysfunction and associated factors reported in the postpartum period. Acta Paulista de Enfermagem, 27(6), 573–578. https://doi-org.proxy-ub.rug.nl/10.1590/1982-0194201400093	Did not compare modes of birth
Durnea C, Carlson V, Khashan A, Kenny LC, O'Reilly BA. Prevalence of pelvic floor dysfunction in primiparous women at 1 year after delivery. International Urogynecology Journal and Pelvic Floor Dysfunction. 2011;22:S74-S5.	Measured pelvic dysfunction not sexual dysfunction
Durnea, C. M., Khashan, A. S., Kenny, L. C., Tabirca, S. S., & O'Reilly, B. A. (2014). The role of prepregnancy pelvic floor dysfunction in postnatal pelvic morbidity in primiparous women. International urogynecology journal, 25(10), 1363–1374. https://doi.org/10.1007/s00192-014-2381-2	Measured pelvic dysfunction not sexual dysfunction
Ejegård, H., Ryding, E. L., & Sjogren, B. (2008). Sexuality after delivery with episiotomy: a long-term follow-up. Gynecologic and obstetric investigation, 66(1), 1–7. https://doi.org/10.1159/000113464	Modes of birth not specified
Faisal-Cury, A., Menezes, P. R., Quayle, J., Matijasevich, A., & Diniz, S. G. (2015). The relationship between mode of delivery and sexual health outcomes after childbirth. The journal of sexual medicine, 12(5), 1212–1220. https://doi.org/10.1111/jsm.12883	Did not use a validated questionnaire
Fauconnier, A., Goltzene, A., Issartel, F., Janse-Marec, J., Blondel, B., & Fritel, X. (2012). Late post-partum dyspareunia: does delivery play a role?. Progres en urologie : journal de l'Association francaise d'urologie et de la Societe francaise d'urologie, 22(4), 225–232. https://doi.org/10.1016/j.purol.2012.01.008	Did not use a validated questionnaire
Fuchs A, Czech I, Dulska A, Drosdzol-Cop A. The impact of motherhood on sexuality. Ginekologia polska. 2021;92(1):1-6.	Did not compare modes of birth
Galbally, M., Watson, S. J., Permezel, M., & Lewis, A. J. (2019). Depression across pregnancy and the postpartum, antidepressant use and the association with female sexual function. Psychological medicine, 49(9), 1490–1499. https://doi.org/10.1017/S0033291718002040	Sexual functioning not measured at ≥ 6 months postpartum
Halouani S, Bettaeib H, Mbarki W, Abidi I, Boufarguine R, Frikha M, et al. Evaluation of the sexual desire disorders in the post-partum period. Journal of Sexual Medicine. 2022;19(5):S232-S3.	Sexual functioning not measured at ≥ 6 months postpartum

Handa VL. Sexual Function and Childbirth. Seminars in Perinatology. 2006;30(5):253-6.	Time when sexual functioning was measured was not specified
Handelzalts JE, Levy S, Peled Y, Yadid L, Goldzweig G. Mode of delivery, childbirth experience and postpartum sexuality. Archives of Gynecology and Obstetrics. 2018;297(4):927-32.	Modes of birth not specified
Hannah, M. E., Whyte, H., Hannah, W. J., Hewson, S., Amankwah, K., Cheng, M., Gafni, A., Guselle, P., Helewa, M., Hodnett, E. D., Hutton, E., Kung, R., McKay, D., Ross, S., Saigal, S., Willan, A., & Term Breech Trial Collaborative Group (2004). Maternal outcomes at 2 years after planned cesarean section versus planned vaginal birth for breech presentation at term: the international randomized Term Breech Trial. American journal of obstetrics and gynecology, 191(3), 917–927. https://doi.org/10.1016/j.ajog.2004.08.004	Did not use a validated questionnaire
Hantoushzadeh S, Shariat M, Rahimi Foroushani A, Ramezanzadeh F, Masoumi M. Sexual satisfaction after child birth: Vaginal versus elective cesarean delivery. Tehran University Medical Journal. 2009;66(12):931-5.	Time when sexual functioning was measured was not specified
Heidari M, Merghati Khoei E, & Valaei N. (2010). Relationship between delivery type and postpartum sexual activity. Advances in Nursing & Midwifery, 20(68), 54.	Abstract and full text not available
Hjorth, S., Kirkegaard, H., Olsen, J., Thornton, J. G., & Nohr, E. A. (2019). Mode of birth and long-term sexual health: a follow-up study of mothers in the Danish National Birth Cohort. BMJ open, 9(11), e029517. https://doi.org/10.1136/bmjopen-2019-029517	Did not use a validated questionnaire
Huang K, Tao F, Liu L, Wu X. Does delivery mode affect women's postpartum quality of life in rural China? Journal of Clinical Nursing. 2012;21(11-12):1534-43.	Did not use a validated questionnaire
Irzan MA, Kayika IPG, Suntoro, Anggraeni TD. Association between mode of delivery and postpartum sexual functioning in primiparous women in Ciptomangunkusumo National General Hospital. Journal of Sexual Medicine. 2015;12:332.	Time when sexual functioning was measured was not specified
Kabakian-Khasholian, T., Ataya, A., Shayboub, R., & El-Kak, F. (2015). Mode of delivery and pain during intercourse in the postpartum period: findings from a developing country. Sexual & reproductive healthcare : official journal of the Swedish Association of Midwives, 6(1), 44–47. https://doi.org/10.1016/j.srhc.2014.09.007	Did not use a validated questionnaire
Karaçam Z. (2008). Effects of episiotomy on sexual function in the postpartum period. Turkish Journal of Research & Development in Nursing, 10(1), 38–46.	Time when sexual functioning was measured was not specified
Irzan MA, Kayika IPG, Suntoro, Anggraeni TD. Association between mode of delivery and postpartum sexual functioning in primiparous women in Ciptomangunkusumo National General Hospital. Journal of Sexual Medicine. 2015;12:332.	Questionnaire used was not specified
Khajehei, M., Doherty, M., Tilley, P. J., & Sauer, K. (2015). Prevalence and risk factors of sexual dysfunction in postpartum Australian women. The journal of sexual medicine, 12(6), 1415–1426. https://doi.org/10.1111/jsm.12901	Did not compare modes of birth
Khajehei M, Ziyadlou S, Safari Rad M, Tabatabaee HR, & Kashefi F. (2009). A comparison of sexual outcomes in primiparous women experiencing vaginal and caesarean births. Indian Journal of Community Medicine, 34(2), 126–130. https://doi-org.proxy-ub.rug.nl/10.4103/0970-0218.51237	Did not use a validated questionnaire
Khalid NN, Jamani NA, Abd Aziz KH, Draman N. The prevalence of sexual dysfunction among postpartum women on the East Coast of Malaysia. Journal of Taibah University Medical Sciences. 2020;15(6):515-21.	Did not compare modes of birth
Kouakou, K. P., Doumbia, Y., Djanhan, L. E., Menin, M. M., & Djanhan, Y. (2015). La sexualité du post-partum. Analyse du vécu dans le couple noir africain [Post-	Not available in English

partum sexuality. Living in black African couple analysis]. Journal de gynecologie, obstetrique et biologie de la reproduction, 44(3), 280–285. https://doi.org/10.1016/j.jgyn.2014.01.001	
Langrová, P., & Vrublová, Y. (2013). Vliv způsobu porodu na ženskou sexualitu [The effect of mode of delivery on woman's sexuality]. Ceska gynekologie, 78(6), 584–588.	Not available in English
Maamri A, Badri T, Boujemla H, Kissi YE. Sexuality during the postpartum period: Study of a population of tunisian women. Tunisie Medicale. 2019;97(5):704-10.	Did not compare modes of birth
Macleod, M., Goyder, K., Howarth, L., Bahl, R., Strachan, B., & Murphy, D. J. (2013). Morbidity experienced by women before and after operative vaginal delivery: prospective cohort study nested within a two-centre randomised controlled trial of restrictive versus routine use of episiotomy. BJOG : an international journal of obstetrics and gynaecology, 120(8), 1020–1026. https://doi.org/10.1111/1471-0528.12184	Did not compare modes of birth
Mahmodiyan A, Kazemi S, Ghojazade M. Comparison of Sexual problems during the first 6 month after normal vaginal delivery and cesarean in nulliparous women referred to health centers of Ramsar in 2014-2015. Iranian Journal of Obstetrics, Gynecology and Infertility. 2017;20(5):7-14.	Not available in English
Makkii, M., & Yazdi, N. A. (2012). Sexual dysfunction during primiparous and multiparous women following vaginal delivery. Tanzania journal of health research, 14(4), 263–268.	Questionnaire used was not specified
Martin, F. Z., Madley-Dowd, P., Ahlqvist, V. H., Jónsson-Bachmann, E., Fraser, A., & Forbes, H. (2022). Mode of delivery and maternal sexual wellbeing: A longitudinal study. BJOG : an international journal of obstetrics and gynaecology, 129(12), 2010–2018. https://doi.org/10.1111/1471-0528.17262	Did not use a validated questionnaire
Mbarki W, Bettaieb H, Frikha M, Abidi I, Halouani S, Boufarguine R, et al. Prevalence of and risk factors associated with vaginal lubrication issues in women at 6 months postpartum. Journal of Sexual Medicine. 2022;19(5):S232.	Measure used for sexual function was not specified or incompletely used
McDonald E, Brown S. Women's sexual health in the first 12 months postpartum. Archives of Women's Mental Health. 2015;18(2):275-6.	Measure used for sexual function was not specified or incompletely used
McDonald E, Woolhouse H, Brown SJ. Consultation about Sexual Health Issues in the Year after Childbirth: A Cohort Study. Birth (Berkeley, Calif). 2015;42(4):354-61.	Did not use a validated questionnaire
McDonald, E., Woolhouse, H., & Brown, S. J. (2017). Sexual pleasure and emotional satisfaction in the first 18 months after childbirth. Midwifery, 55, 60–66. https://doi.org/10.1016/j.midw.2017.09.002	Did not use a validated questionnaire
Molin, B., Sand, A., Berger, A. K., & Georgsson, S. (2020). Raising awareness about chronic pain and dyspareunia among women - a Swedish survey 8 months after childbirth. Scandinavian journal of pain, 20(3), 565–574. https://doi.org/10.1515/sjpain-2019-0163	Did not use a validated questionnaire
Nikpoor P, De Souza AM, Karmakar D, Dwyer PL, Thomas E, Murray C. Comparison of female sexual function with mode of delivery at 12months and 5 years: A prospective longitudinal cohort study from atertiary hospital. Female Pelvic Medicine and Reconstructive Surgery. 2019;25(5):S158-S9.	Did not compare modes of birth
Oboro, V. O., & Tabowei, T. O. (2002). Sexual function after childbirth in Nigerian women. International journal of gynaecology and obstetrics: the official organ of the International Federation of Gynaecology and Obstetrics, 78(3), 249–250. https://doi.org/10.1016/s0020-7292(02)00151-0	Abstract and full text not available
O'Malley, D., Higgins, A., Begley, C., Daly, D., & Smith, V. (2018). Prevalence of and risk factors associated with sexual health issues in primiparous women at 6 and 12 months postpartum; a longitudinal prospective cohort study (the MAMMI study). BMC pregnancy and childbirth, 18(1), 196. https://doi.org/10.1186/s12884-018-1838-6	Did not use a validated questionnaire

Prado DS, Mendes RB, Gurgel RQ, Barreto IDC, Cipolotti R, Gurgel RQ. The influence of mode of delivery on neonatal and maternal short and long-term outcomes. Revista de saude publica. 2018;52:95.	Questionnaire used was not specified
Qian, R., Chen, Z., Tang, L., & Zhang, W. (2016). Postpartum adverse effects and sexual satisfaction following cesarean delivery in Beijing. International journal of gynaecology and obstetrics: the official organ of the International Federation of Gynaecology and Obstetrics, 132(2), 200–205. https://doi.org/10.1016/j.ijgo.2015.07.010	Did not use a validated questionnaire
Rezaei, N., Azadi, A., Sayehmiri, K., & Valizadeh, R. (2017). Postpartum Sexual Functioning and Its Predicting Factors among Iranian Women. The Malaysian journal of medical sciences : MJMS, 24(1), 94–103. https://doi.org/10.21315/mjms2017.24.1.10	Sexual functioning not measured at ≥ 6 months postpartum
Rosen, N. O., Dawson, S. J., Binik, Y. M., Pierce, M., Brooks, M., Pukall, C., Chorney, J., Snelgrove-Clarke, E., & George, R. (2022). Trajectories of Dyspareunia From Pregnancy to 24 Months Postpartum. Obstetrics and gynecology, 139(3), 391–399. https://doi.org/10.1097/AOG.0000000000004662	Measure used for sexual function was not specified or incompletely used
Rouhi, M., Vizheh, M., Rouhi, L., Esmaili, H., Vaziri, L., & Gherekhlou, Z. F. (2016). Postpartum morbidities in Iranian women 5 years after childbirth: A longitudinal study. British Journal of Midwifery, 24(4), 268–274. https://doi-org.proxy-ub.rug.nl/10.12968/bjom.2016.24.4.268	Did not compare modes of birth
Safarinejad MR, Kolahi AA, Hosseini L. The effect of the mode of delivery on the quality of life, sexual function, and sexual satisfaction in primiparous women and their husbands. Journal of Sexual Medicine. 2009;6(6):1645-67.	Time when sexual functioning was measured was not specified
Saleh DM, Hosam F, Mohamed TM. Effect of mode of delivery on female sexual function: A cross-sectional study. Journal of Obstetrics and Gynaecology Research. 2019;45(6):1143-7.	Sexual functioning not measured at ≥ 6 months postpartum
Saotome T, Suganuma N. Factors associated with sexuality in postpartum couples. Journal of Sexual Medicine. 2017;14(5):e259.	Did not compare modes of birth
Saotome TT, Yonezawa K, Suganuma N. Sexual Dysfunction and Satisfaction in Japanese Couples During Pregnancy and Postpartum. Sex Med. 2018;6(4):348-55.	Did not compare modes of birth
Saurel-Cubizolles MJ, Romito P, Lelong N, Ancel PY. Women's health after childbirth: A longitudinal study in France and Italy. British Journal of Obstetrics and Gynaecology. 2000;107(10):1202-9.	Did not use a validated questionnaire
Shirvani, M. A., Nesami, M. B., & Bavand, M. (2010). Maternal sexuality after child birth among Iranian women. Pakistan journal of biological sciences : PJBS, 13(8), 385–389. https://doi.org/10.3923/pjbs.2010.385.389	Modes of birth not specified
Soljačić Vraneš H, Djaković I, Vraneš H, Vrljičak M, Kraljević Z. Changes in Sex Life after Childbirth. Psychiatria Danubina. 2019;31:847-50.	Questionnaire used was not specified
Szöllősi K, Szabó L. PS-2-3 Postpartum Female Sexual Dysfunctions in Hungary: A Prospective Longitudinal Study. Journal of Sexual Medicine. 2020;17(6):S126.	Modes of birth not specified
Szöllosi K, Szabó L. Postpartum female sexual problems and related conditions in Hungary a cross sectional study. Journal of Perinatal Medicine. 2019;47:eA423.	Sexual functioning not measured at ≥ 6 months postpartum
Thompson, J. F., Roberts, C. L., Currie, M., & Ellwood, D. A. (2002). Prevalence and persistence of health problems after childbirth: associations with parity and method of birth. Birth (Berkeley, Calif.), 29(2), 83–94. https://doi.org/10.1046/j.1523-536x.2002.00167.x	Did not use a validated questionnaire

Triviño-Juárez, J. M., Romero-Ayuso, D., Nieto-Pereda, B., Forjaz, M. J., Oliver-Barrecheguren, C., Mellizo-Díaz, S., Avilés-Gámez, B., Arruti-Sevilla, B., Criado-Álvarez, J. J., Soto-Lucía, C., & Plá-Mestre, R. (2018). Resumption of intercourse, self-reported decline in sexual intercourse and dyspareunia in women by mode of birth: A prospective follow-up study. Journal of advanced nursing, 74(3), 637–650. https://doi.org/10.1111/jan.13468	Did not use a validated questionnaire
Unal Aslan KS, Cetinkaya F, Gozuyesil E. Adolescent mothers' postpartum sex life quality: A cross-sectional study. Konuralp Tip Dergisi. 2020;12(2):282-9.	Sexual functioning not measured at ≥ 6 months postpartum
Woolhouse H, McDonald E, Brown SJ. Changes to sexual and intimate relationships in the postnatal period: Women's experiences with health professionals. Australian Journal of Primary Health. 2014;20(3):298-304.	Did not compare modes of birth
Xu XY, Wang HY, Su L, Peng B, Yao ZW. Women's sexual health after delivery and its related influential factors. Journal of Clinical Rehabilitative Tissue Engineering Research. 2007;11(17):3438-42.	Did not compare modes of birth
Xu, X. Y., Yao, Z. W., Wang, H. Y., Zhou, Q., & Zhang, L. W. (2003). Zhonghua fu chan ke za zhi, 38(4), 219–222.	Questionnaire used was not specified
Yee L, Nakagawa S, Kaimal A, Kuppermann M. Postpartum sexual functioning and mode of delivery in a diverse population of women. American Journal of Obstetrics and Gynecology. 2012;206(1):S155.	Poster/conference abstract of paper that was already included
Zgliczynska M, Zasztowt-Sternicka M, Szymusik I, Rowicki L, Majewska A, Kosinska-Kaczynska K. 065 How Does Selected Factors Associated with Pregnancy and Childbirth Affect Women's Sexuality? Female Sexual Function Index before Pregnancy and After Delivery. Journal of Sexual Medicine. 2019;16(6):S24.	Did not compare modes of birth
Zivkovic K. HP-02-005 The role of lateral episiotomy on the emergence of sexual dysfunction in primiparas. Journal of Sexual Medicine. 2019;16(5):S34.	Did not compare modes of birth
